# Stability and safety key factors of the oncolytic protoparvovirus H-1 from manufacturing to human application

**DOI:** 10.1007/s00253-023-12521-4

**Published:** 2023-05-20

**Authors:** Veronika Frehtman, Daniel Wohlfarth, Marcus Müller, Ottheinz Krebs, Barbara Leuchs

**Affiliations:** German Cancer Research Center, Tumor Virology, Im Neuenheimer Feld 280, 69120 Heidelberg, Germany

**Keywords:** Protoparvovirus H-1PV, Formulation, Stability, Environmental safety, Deactivation, Drug product

## Abstract

**Abstract:**

The oncolytic rodent protoparvovirus H-1PV has been successfully used in phase I/II clinical trials to treat recurrent glioblastoma multiforme and pancreatic cancer. The present work focuses on the stability and environmental safety of the H-1PV drug product from production up to its use in patients. We identified hold-steps in manufacturing for up to 3 months and showed 7-years stability for the optimal product formulation. Stress testing via UV, temperature, and pH also determined that the drug product is stable. De- and rehydration for lyophilization simulation are possible without infectious virus loss. Furthermore, we prove in-use stability for 4 days at room temperature and show no virus adsorption to injection devices, guaranteeing the correct administration dose. Iodixanol in the formulation, resulting in high viscosity, protects H-1PV against UV and some disinfectants. Nonetheless, H-1PV is depleted with rapid heat deactivation, autoclavation, and nanofiltration. Assessment of chemical disinfectants that are currently recommended by the Robert Koch-Institute demonstrated that ethanol-based hand disinfectants are not effective; however, aldehyde-based disinfectants for surfaces and instruments demonstrate sufficient H-1PV deactivation in aqueous formulations by 4 to 6 log_10_. With these results, we could establish a specific hygiene plan for all involved facilities from manufacturing to patient application. Overall, using 48% Iodixanol in Visipaque/Ringer as a drug formulation stabilizes H-1PV infectivity over years and protects against virus loss from short-term UV, low pH, and temperature exposure.

**Key points:**

*• Optimal formulation of drug product protects the H-1PV protoparvovirus against UV, temperatures up to 50 °C, and low pH (> 1.25), stabilizing the virus during manufacturing, storage, transport, and application.*

*• H-1PV is stable during in-use and does not adsorb to injection devices during patient administration.*

*• Hygiene plan for H-1PV with physicochemical methods has been established.*

**Supplementary Information:**

The online version contains supplementary material available at 10.1007/s00253-023-12521-4.

## Introduction

During the process of developing a drug product and its successful application, the stability of the drug is crucial at any given production or application step, as are safety aspects concerning the patient and the environment. Therefore, stability, environmental safety, and deactivation of a drug product must be defined regarding the manufacturing, pharmaceutical, and clinical facility, associated analytical laboratories, during transport, as well as for all involved. The oncolytic virus drug ParvOryx consists of wild-type parvovirus H-1PV. It belongs to the genus *Protoparvovirus* (Cotmore et al. 2014), is a small non-enveloped virus of 25 nm in diameter, and contains a single-stranded DNA genome about 5 kb in length (Faisst et al. 1998; Halder et al. 2012; Hanson and Rhode 1991). In nature, rats are the hosts of H-1PV, the virus is shed through feces and transmission occurs via oronasal route in animals (Bretscher and Marchini 2019). The clinical trial material (CTM) was successfully used in a clinical phase I/II dose-escalation study in patients with recurrent glioblastoma multiforme ParvOryx01 (Geletneky et al. 2017) and phase I/II clinical trial in patients with inoperable metastatic pancreatic ductal adenocarcinoma ParvOryx02 (Hajda et al. 2021). These studies demonstrated the safety and immunogenic activity of the drug, which is administered either by intravenous (i.v.) or local intratumoral (i.t.) injection. The virus shedding is primarily through feces, as expected from animal experiments. Target distribution of the virus inoculum and a loss of active substance during storage and application constitute challenges for these procedures, termed *in-use*. In particular, when a catheter is used, the virus is distributed along the catheter track due to an area of diminished resistance along this path through the tissue. This phenomenon is known as backflow and could result in less active substance being delivered to the target tissue.

An optimal formulation of a drug can help to overcome such application challenges and accommodate all critical steps of a drug from manufacturing to application. It also plays a crucial role in the stability and deactivation assurance of the drug.

We demonstrate here important steps for virus stability as well as methods to inactivate the virus to ensure environmental safety. In the H-1PV production process (Leuchs et al. 2016) several significant changes were implemented in the last few years, two of which have been published: first, production of the H-1PV virus in a bioreactor with macrocarriers (Wohlfarth et al. 2021); second, changes in the downstream purification process with chromatographic purification (Leuchs et al. 2017). These changes have helped to optimize quality, quantity, reproducibility, and scalability of the up- and downstream process, respectively.

All the data reported here were generated during the clinical trials and while developing the process under laboratory conditions except for the data on the stability of clinical batches produced under GMP conditions.

## Material and method

### Drug production and formulation

NB-324 K human newborn kidney cells (Tattersall and Bratton 1983) were cultured at 37 °C in VP-SFM™ medium (Thermo Fisher, NY, USA) with 5% fetal bovine serum (FBS, Biowest, Nuaille, France) in a 5% CO_2_ atmosphere. For H-1PV production, cells were seeded in the iCELLis^®^ nano bioreactor system (Pall, Port WA, USA) at a concentration of 5E3 cells/cm^2^ or in 10-layer CellSTACK^®^ (CS; Corning, Wiesbaden, Germany) according to Leuchs et al. (2016) in VP-SFM™ medium supplemented with 2% FBS. The cells in the iCELLis^®^ nano bioreactor system were infected on day 5 and the cells in CS were infected on day 3 with a virus stock of wild-type H-1PV. The virus stock was produced via transfection (Kestler et al. 1999) of NB-324K cells and subsequently propagated by two rounds of infection. Here, a multiplicity of infection (MOI) of 0.01 was applied during 100% medium exchange to VP-SFM™ medium supplemented with 1% FBS followed by a medium exchange without FBS 2 days after infection. The cells were harvested after 4 days of incubation with Tris-based (Trizma^®^ hydrochloride; Sigma-Aldrich Co, St. Louis, USA) harvest buffer, followed by 0.2 μm clarification. Thereafter, the cells were treated with DNAse and buffer exchanged to chromatography buffer with tangential flow filtration. The chromatography and buffer exchange to Ringer solution (AlleMan Pharma GmbH, Reutlingen, Germany) was followed by formulation in Visipaque™ (48% Iodixanol in Visipaque/Ringer) according to Leuchs et al. (2017). Visipaque™ 320 (contains 65.2% Iodixanol; GE Healthcare Europe GmbH, Freiburg, Germany) was diluted to 48% Iodixanol with Ringer solution. The refraction index of 48% Iodixanol in Visipaque/Ringer solution is 1.41 ± 0.005 and must be verified in this final formulation (DP-F). For i.v. injection, the drug product was diluted to 10% Iodixanol with Ringer solution (DP-i.v.). The GMP batches were produced by IDT Biologika, Germany.

### Formulation for stability and deactivation studies

H-1PV in DP-F and DP-i.v. formulations was used for the majority of stability studies since these formulations are used in clinic. The virus was also formulated in pure Ringer solution (H-1PV-RIN) as it is an intermediate formulation after the 2nd buffer exchange (Leuchs et al. 2017) or in Tris-based buffer (H-1PV-VTE), which was previously used as harvest buffer (Leuchs et al. 2016). The buffer composition is summarized in Table 1. All deactivation studies were performed with H-1PV in DP-F. UV deactivation and chemical deactivation studies with Incidin™ Rapid and Sekusept™ aktiv were additionally done with DP-i.v., H-1PV-RIN, and H-1PV-VTE formulations.

**Table 1:** Overview of different formulation compositions used in manufacturing and clinical application

Formulation	Buffer composition	~pH	Abbreviation	Clinical use	Application
Drug product	48% Iodixanol in Visipaque/Ringer	7.3	DP-F	Intrametastatic, intratumoral	Catheter
				Intracerebral	Direct injection
Intravenous application	10% Iodixanol in Visipaque/Ringer	7.2	DP-i.v.	Intravenous	Cardiac catheterization
Ringer	Ringer	5–7	H-1PV-RIN	None	None
VTE	50 mM Tris-EDTA	8.7	H-1PV-VTE	None	None

### Quantification of H-1PV infectious titer

The infectious titer for H-1PV was determined by performing a plaque formation assay. Titer is expressed as plaque-forming units (PFU) per milliliter. The precision of the plaque assay is defined by the standard deviation (European-Medicines-Agency 1995). Our quality control determination repeated > 40 times shows a standard deviation of 0.33 log_10_ (PFU/ml). This PFU deviation is the accepted range for plaque variation. Infectious units (IU) titer was analyzed by DNA hybridization assay after infection, where titer is expressed in IU per milliliter (see Leuchs et al. (2016) for both methods description). All infectious titers were analyzed by PFU assay. Only in the deactivation study some infectious titers were measured via IU assay (Table 2).

### Stability testing

For hold-steps stability testing, H-1PV was stored in harvest buffer, chromatography equilibration buffer after 1st TFF, and in elution buffer after chromatography for 3 months at 4 °C. After establishing chromatographic purification of the H-1PV (Leuchs et al. 2017), H-1PV batches formulated in DP-F were stored at −20 °C and analyzed after 12, 24, 48, 36, 48, and 60 months. The GMP batches were stored up to 7 years at < −65° C at IDT Biologika, Germany.

### UV, pH, and temperature stability

Stress tests were performed with UV, pH, and temperature. Detailed UV treatment is described under the section “*Physical deactivation*.” The pH stability was tested by adjusting the H-1PV in DP-F to a pH of 1, 1.25, 1.5, 1.75, 2, 3, and 4 with HCl for 30 min. After 30 min the H-1PV/HCl solution was neutralized with VP-SFM™ medium containing 5% FBS. For temperature stress tests, H-1PV in DP-F and DP-i.v. was stored at 4 °C before being placed at 37 °C and 50 °C for 4 h, 8 h, 24 h, 48 h, 72 h, and 96 h.

### De- and rehydration stability

Lyophilization of H-1PV in DP-F was simulated by dehydration of H-1PV in four different concentrations with an evaporator (Jouan RC 10-10 Vacuum Concentrator Centrifugal Evaporator, Thermo Electron Corporation, France) at 37 °C overnight. For reconstitution, the same amount of water used for injection (B.Braun, Germany) was added and incubated for 2 h at 37 °C.

### In-use stability

The ParvOryx drug in DP-F and DP-i.v. was prepared at the clinical pharmacy for ParvOryx01 and ParvOryx02 patient applications and stored at room temperature (RT) of 21 ± 0.5 °C for up to 4 days.

### Adsorption of final injection system

The H-1PV adsorption to the injection sets such as needle, syringe, and catheter in combination with diverse connectors used for ParvOryx01 and ParvOryx02 clinical trials (mainly made, for example, of polypropylene, polyethylene, polyurethane, or polycarbonate) was assessed. All injection materials were assembled as used in the trials and circulated 10 times with H-1PV in DP-F or the corresponding DP-i.v. formulation, or remaining virus was taken from the device after administration to the patient and then analyzed.

### Physical deactivation

For UV deactivation, we employed a 254-nm-wavelength UV lamp (Herolab NU-4, Germany), resulting in 240 μW/cm^2^. The treatment of 500-μl virus in an open 35 mm cell culture dish was repeated four times for 2 min each with 5-min pauses. The UV intensity was controlled with a UVA/UVC Light Meter SDL470 (EXTECH Instruments, USA). Depletion of H-1PV in the DP-F formulation via nanofiltration was evaluated by using a 30-kDa cut-off Vivaspin^®^ filter (Sartorius AG, Germany). Heat deactivation was performed on H-1PV in DP-F at 99 °C for 10 min with Eppendorf Thermomixer comfort heating block (Eppendorf, Germany), whereas autoclavation was at 121 °C for 20 min with a MMM autoclave (Münchner Medizin Mechanik GmbH, Germany).

### Chemical deactivation

Three ethanol-based disinfection solutions (Sterillium^®^ Virugard from Paul Hartmann AG, Germany, Spitacid™, and Skinman™ complete from Ecolab GmbH, Germany) were tested for hand disinfection against H-1PV. For this, nine parts of disinfection solution were combined with one part of H-1PV solution in DP-F and incubated for the times outlined in Table 2 at RT. To assess the surface and instrument disinfectants, six chemical solutions were tested: 1. Lysoformin^®^ (Lysoform Dr. Hans Rosemann GmbH, Germany); 2. Korsolex^®^ basic (Paul Hartmann AG, Germany); 3. Incidin™ Rapid; 4. Sekusept™ aktiv (Ecolab GmbH, Germany); 5. Glutaraldehyde 25% (Millipore, Germany); and 6. Sodium hydroxide (Roth, Germany). All chemicals were diluted to the final concentrations with H-1PV solution in DP-F. Additionally, Incidin™ Rapid and Sekusept™ aktiv were used for H-1PV deactivation in DP-i.v., H-1PV-RIN, and H-1PV-VTE formulations. Incubation times at RT of 22 ± 1 °C are presented in Table 3. The disinfection procedure was carried out immediately before titrating the infectious virus. For neutralization, the virus/disinfectant solution was diluted in MEM medium with 5% FBS.

### Statistical analysis

We performed a *t*-test (one-tailed, dependent, *p* < 0.05) with the Excel Add-in Analysis ToolPak (Microsoft Corporation, Redmond, USA) for all stability data. Exceptions are the in-use stability and GMP batches stability data, where the *t*-test was not applicable. Here, the precision of the PFU assay with a deviation of ≤ 0.33 log_10_ (PFU/ml) was set as boundary for statistical significance. Only statistically insignificant data or outliers are mentioned in the result. For deactivation studies the reduction factor is presented as the difference between the virus titer before and after physico-chemical treatment. This difference is given as log_10_ reduction infectious titer with standard deviation. A reduction in viral titers of ≥ 4 log_10_ ± 0.33 (inactivation ≥ 99.99%) is defined as a significant proof of virucidal activity (Rabenau et al. 2020).

## Results

**Fig. 1:**
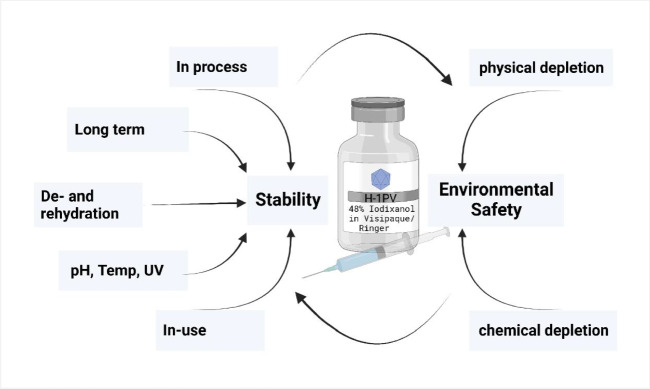
Key factors analyzed regarding stability and depletion of virus for environmental safety that are influenced by the formulation. The figure was created using BioRender.com

The formulation of H-1PV drug products strongly influences H-1PV stability during the process and plays an important role for environmental, in-use, and long-term stability. To ensure environmental safety, a cleaning and disinfection protocol must be established. The diagram in Fig. 1 illustrates these influences. Thus, if a formulation is changed, these key aspects must be reevaluated.

### Formulation

For the final formulation of the drug product (DP), we selected 48% Iodixanol in Visipaque /Ringer solution (DP-F). This formulation shows a viscosity similar to that of blood of approximately 4 mPa*s at 37 °C. For intratumoral treatment, the drug was administered directly into the tumor and for the i.v. route 10% Iodixanol diluted in Visipaque/Ringer (DP-i.v.) was injected (Table 1).

### Hold-steps stability during manufacturing and final drug product stability

**Fig. 2:**
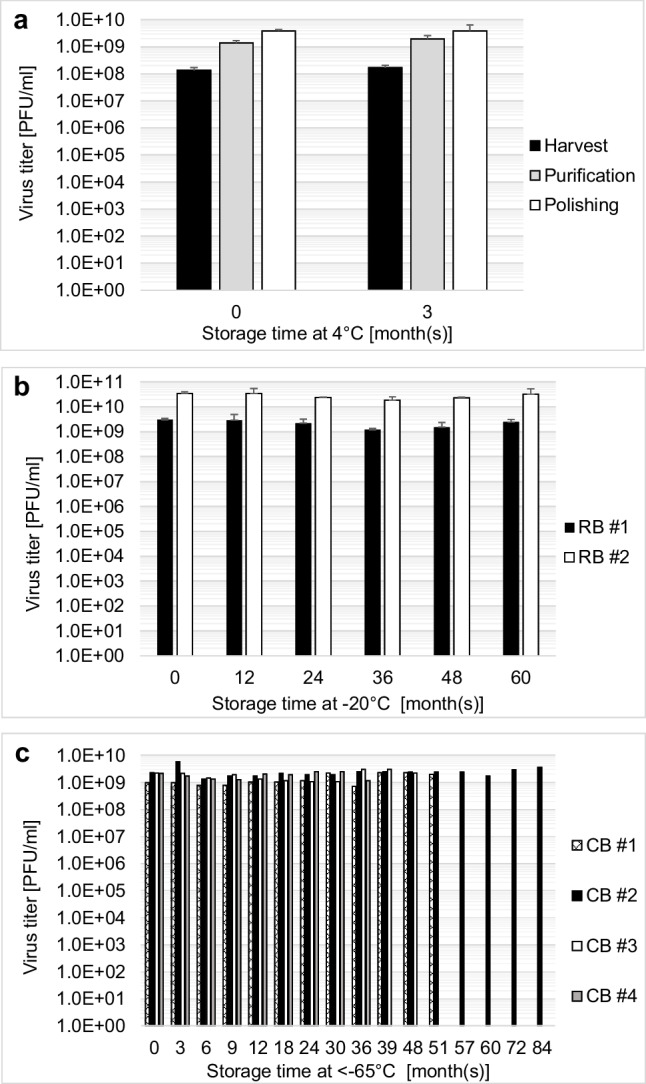
Short- and long-term stability of H-1PV. **a** Stability study was performed for three different process steps at 4 °C, after harvest, during purification, and during polishing. **b** Long-term stability of DP-F at −20 °C. Stability study was performed with two research-grade batches (RB) with a virus titer of 2.0E9 PFU/ml (RB #1) and 2.0E10 PFU/ml (RB #2). **c** Long-term stability up to 7 years at < −65 °C in DP-F of four GMP-produced clinical batches (CB #1–4). The *t*-test for Fig. 2a and b demonstrated statistically significant stability, excluding the weak outlier at 36 months for RB #1 at Fig. 2b(> 0.33 < 0.4 log_10_ (PFU/ml)). GMP batches are stable in Fig. 2c using plaque precision criteria

During the drug manufacturing process, foreseeable (i.e., incubation times and measurement time for analytics and storage) and unforeseeable (i.e., device failure, manpower shortage, and disruptions in supply chain) stops may occur. Hold-steps can compensate for such stops in the process. However, pre-defined formulation, storage temperature, and duration with assured product stability are required. We developed a novel up- (Wohlfarth et al. 2021) and downstream process (Leuchs et al. 2017) to improve the quantity and quality and up-scale the process for market release of the drug. Suitable hold-steps with stable virus titers at 4 °C for up to 3 months were identified in real-time stability study after harvest, purification via the 1st TFF including buffer exchange and polishing via chromatography for three production batches (Fig. 2a). During the purification and polishing the virus is concentrated resulting in higher titer, while the majority of protein (99.9%) and DNA (98.0%) impurities are depleted during these steps (data not shown).

By applying the selected hold-steps, a flexible manufacturing schedule can be achieved and production consistency can be maintained at laboratory scale due to the low standard deviation of the virus titer in all manufacturing steps. Additionally, two individual batches of drug product in the final formulation manufactured with virus titers simulating lower (RB #1) and upper virus titer (RB #2) were produced using the novel process and subsequently stored at −20 °C. With both research batches, a real-time stability study was performed over the course of 5 years, showing constant virus titers excluding the weak outlier at 36 months for RB#1 (> 0.33< 0.4 log10 (PFU/ml)) in the middle of the stability series (Fig. 2b). Furthermore, we demonstrated that the H-1PV produced under GMP conditions for the phase I/II clinical trial and formulated in 48% Iodixanol in Visipaque/Ringer was stable for over 7 years at < −65 °C (Fig. 2c).

### Stability of drug product via stress test

**Fig. 3:**
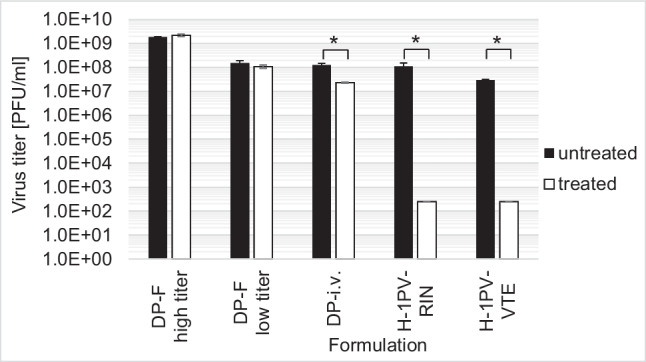
UV stability of H-1PV in different formulations with various virus titers. Titer untreated and after UV treatment with 240 μW/cm^2^ for 2-min and 5-min breaks (repeated four times) represent in total a 10-min deactivation with 254 nm and 240 μW/cm^2^. *p < 0.05

**Fig. 4:**
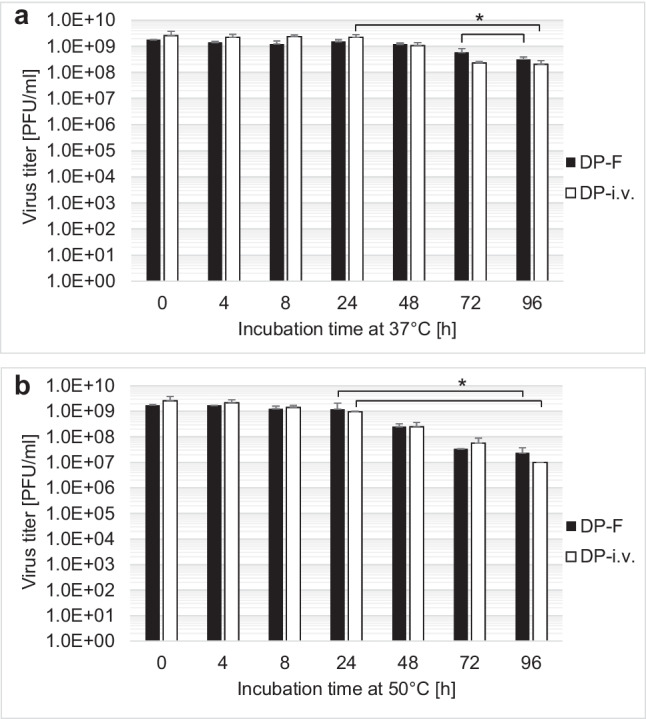
Temperature stability of H-1PV at 37 °C (**a**) and at 50 °C (**b**) in either DP-F and DP-i.v. formulation for up to 96 h. *p < 0.05

For the stress test, we assessed the effects of UV, pH, and temperature treatment on H-1PV in the drug product and other formulations. Iodixanol stabilizes and protects H-1PV against UV radiation. This could be demonstrated by reducing the Iodixanol concentration to 10% Iodixanol in Visipaque/Ringer (DP-i.v.) and to 100% Ringer (H-1PV-RIN) and Tris-EDTA buffer (H-1PV-VTE). At 10% Iodixanol, UV reduced the titer by 0.7 log_10_. Virus in Ringer and VTE buffer shows a 5 log_10_ titer reduction (Fig. 3). H-1PV is stable under low pH conditions (pH ≥ 1.25) for up to 30 min (supplemental data, Fig. S1). Temperature stability in the final formulation of 48% Iodixanol in Visipaque/Ringer and 10% Iodixanol in Visipaque/Ringer was evaluated to ensure that temperature variations during transport and from pharmacy to patient does not challenge the stability of the drug. H-1PV in DP-F demonstrates stability up to 72 h at 37 °C and up to 24 h at 50 °C. H-1PV in DP-i.v. is stable up to 24 h at both 37 °C and 50 °C. Beyond these time points, a successive reduction in the infectious titer is observed (Fig. 4a and b).

### De- and rehydration stability

**Fig. 5:**
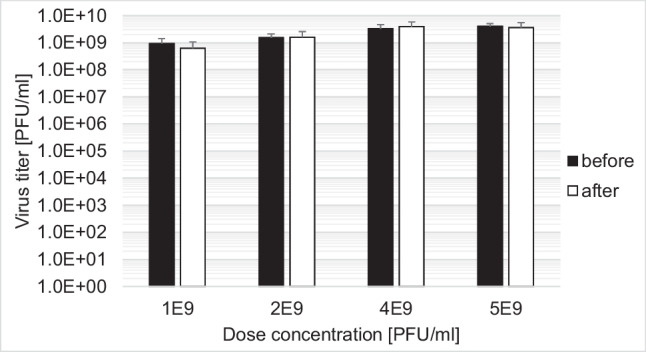
Stability of H-1PV in DP-F after dehydration and reconstitution in the same starting volume with water. Titers were measured before dehydration and after reconstitution demonstrating significant stability

Lyophilization would offer an advantage for transport of the drug product. Therefore, we simulated lyophilization by dehydration and reconstitution of the drug product in DP-F. We tested concentrations of 1E9, 3E9, 4E9, and 5E9 PFU/ml. H-1PV remained stable and no reduction in the infectious titer was detected after de- and rehydration (Fig. 5).

### In-use stability

**Fig. 6:**
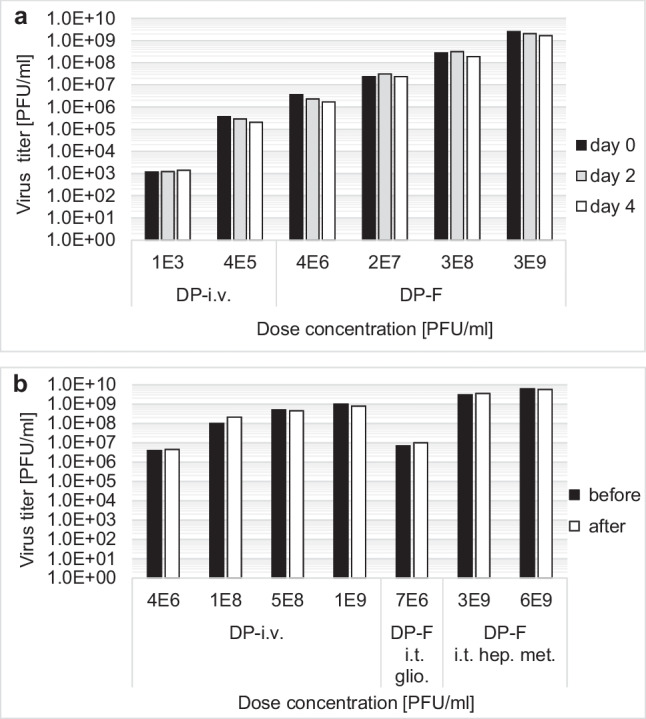
**a** In-use stability of H-1PV drug products with different doses over 4 days at 21 ± 0.5 °C. Titers are measured on day 0, day 2, and day 4 either for i.v. injection (DP-i.v.) or intratumoral injection (DP-F) indicating significant stability. **b** H-1PV adsorption to used injection sets in clinical trials was tested for i.v. administration in DP-i.v. or i.t. injection of glioblastoma tumor (glio.) and hepatic metastasis (hep. met.) in DP-F. Titers were measured before and after administration demonstrating significant stability

It is important to demonstrate the stability of the drug during in-use. For this, the ParvOryx drug used in clinical studies was tested in-use at 21 ± 0.5 °C for up to 4 days. The stability of different doses in DP-F and DP-i.v. formulations could be successfully demonstrated, as seen in Fig. 6a.

For clinical application, it is also important to demonstrate that H-1PV does not interact with any material used for injection. Here, different doses of H-1PV in DP-i.v. and DP-F formulations were prepared in their respective injection systems for use in ParvOryx01 and ParvOryx02 clinical trials. The infectious titer was analyzed before and after administration or administration simulation via the injection system. No reduction in virus titer due to adsorption was observed so that all patients received the planned dose (Fig. 6b).

### Environmental safety aspects for human use

Up to now, no effects on health or any detrimental long-term or delayed effects of parvovirus H-1 have been reported in humans in the ParvOryx01 and ParvOryx02 studies (Geletneky et al. 2017; Hajda et al. 2021). Furthermore, we indirectly observed that H-1PV is not easily transmitted through the oronasal route or via human contamination. For this, blood serum of a study nurse working under the clinical conditions during clinical trials and of laboratory staff (*n* = 8), who have been working routinely with H-1PV for many years under biosafety level 2 conditions, was analyzed. No antibody response was measured in the hemaglutination inhibition assay (performed at Labor Enders, Germany) (see Supplemental Data, Table S1).

Nevertheless, specific hygiene procedures are necessary for manufacturing, preparation of the final patient injection, and for the hospital to guarantee environmental safety. Therefore, a deactivation protocol must be established either with physical or chemical methods.

### Physical depletion of infectious H1-PV

**Fig. 7:**
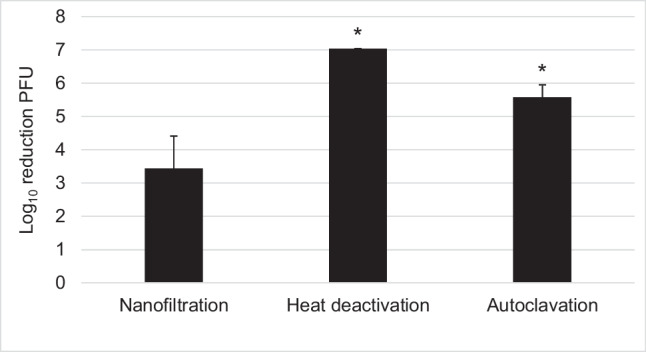
Physical depletion of H-1PV by nanofiltration with 30-kDa Vivaspin^®^, heat deactivation at 99 °C for 10 min, or autoclaving at 121 °C for 20 min. *≥ 4 log_10_ ± 0.33

For physical depletion, we tested in addition to UV deactivation, heat inactivation, autoclavation (steam sterilization) and nanofiltration. For nanofiltration, we utilized the known molecular size of H-1PV to select a suitable and commercially readily available filtration system with a cut-off of 30 kDa to retain the larger virus. Nanofiltration with a 30-kDa cut-off resulted in a mean infectious titer reduction of 3.4 log_10_. UV deactivation is effective for the aqueous solution; however, H1-PV is protected quite well against UV irradiation in the DP-F and DP-i.v. formulations (see Fig. 3). To physically inactivate H-1PV in DP-F by temperature, we assessed rapid heat deactivation at 99 °C for 10 min and autoclavation at 121 °C for 20 min. Rapid heat deactivation and autoclavation are both safe and reliable methods to inactivate virus as the infectious virus titer is reduced by more than 5 log_10_ (Fig. 7).

### Chemical deactivation of infectious H-1PV

**Fig. 8:**
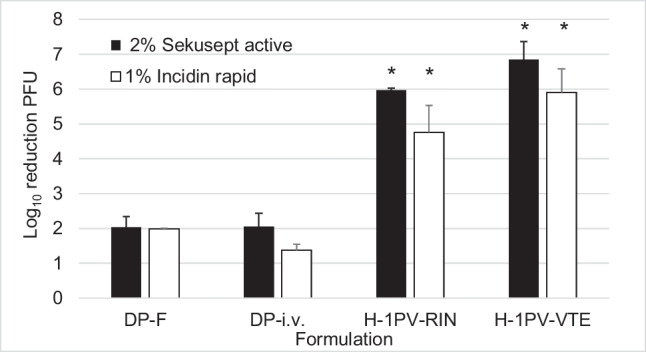
Deactivation of H-1PV with the oxidizer 2% Sekusept™ aktiv for 4 h and with the aldehyde 1% Incidin™ Rapid for 1 h at room temperature in different formulations. *≥ 4 log_10_ ± 0.33

For chemical inactivation of a small, non-enveloped virus, compounds such as alcohols, oxidizers, aldehydes, acids, alkaline, or quaternary ammonium compounds are commonly used. For hand hygiene, compounds such as alcohol are regularly used whereas aldehydes, oxidizers, alkaline, or quaternary ammonium compounds are employed to disinfect medical instruments and surfaces. Solutions that are currently recommended by the Robert Koch-Institute (RKI) Germany for hand hygiene, surface, and instrument disinfection (Robert-Koch-Institut 2017, 2022) were reviewed and compared to the German Cancer Research Center (DKFZ) hygiene plan. Disinfection products recommended for hands, surfaces, and instruments were selected and assessed using H-1PV in 48% Iodixanol in Visipaque/Ringer. We also included two noncommercial solutions in our experiments, for example, glutaraldehyde, which is recommended for virus inactivation when preparing electron microscopy (ELMI) samples (Rodgers et al. 1985), and sodium hydroxide solution, commonly required for cleaning-in-place (CIP) procedures of downstream devices. Different downstream systems require different NaOH concentrations for CIP procedures. Therefore, we assessed the lowest (0.05 M) and the highest (1 M) recommended concentrations with an incubation time of 15 min. The results for glutaraldehyde and sodium hydroxide were sufficient at 4.0 ± 0.7 and 5.9 ± 0.2 (0.05 M NaOH) or 6.5 ± 0.7 (1 M NaOH) log_10_ infectious titer reduction, respectively. However, all three ethanol-based hand disinfectants tested failed to inactivate H-1PV (Table 2). H-1PV inactivation achieved with various aldehyde-based products demonstrate infectious titer reductions of 2 to 6 log_10_. Peracetic acid-based oxidizer Sekusept™ aktiv does not demonstrate sufficient virucidal properties, inactivating only 2.0 ± 0.3 log_10_ H-1PV infectious titer in DP-F (see Table 3). The effectiveness of a disinfection solution is influenced by the formulation, temperature, contact time, or pH. Here, we assessed four different formulations (DP-F, DP-i.v., H-1PV-RIN, and H-1PV-VTE) with Incidin™ Rapid and Sekusept™ aktiv, since the evaluation of these compounds with DP-F formulation delivered unexpectedly low deactivation results. As seen in Fig. 8, the DP-F and DP-i.v. formulations demonstrate protection against H-1PV inactivation in at least 10% Iodixanol in Visipaque/Ringer (DP-i.v.). Consequently, both aldehyde-based and peracetic acid-based compounds could only deactivate approximately 2 log_10_ of H-1PV infectious titer in Iodixanol-based formulations. H-1PV is more susceptible to deactivation in aqueous formulations without Iodixanol, demonstrating a more than 4 log_10_ reduction in infectious titer in Ringer and one of approximately 6 log_10_ in Tris buffer.

**Table 2:** Hand hygiene solution tests by mixing 9 parts of hand disinfection and 1 part virus solution in 48% Iodixanol in Visipaque/Ringer

Solution	Compound	Active component	Incubation time [min]	Log_10_ reduction infectious titer
Sterillium® virugard	Alcohol	95% ethanol	15	0.4 ± 0.4
Spitacid™		46.0% ethanol, 27.0% 2-propanol, 1.0% benzyl alcohol	15	0.3 ± 0.5
Skinman™ complete		89% ethanol, 1% butan-2-on	2	0.7 ± 0.6

**Table 3:** Deactivation of H-1PV in 48% Iodixanol in Visipaque/Ringer with surface and instrument disinfectants, *≥ 4 log_10_ ± 0.33

Solution	Compound	Active component	Concentration of solution	Incubation time [min]	log_10_ reduction infectious titer
Lysoformin^®^	Aldehyde	6% formaldehyde, 1.8% glutaral	10%	60	3.0 ± 0.0
Incidin™ rapid		9.8% glutaral, 5% benzalkonium chloride, 5% didecyldimethylammonium chloride	1%	60	2.0 ± 0.0
Korsolex^®^ basic*		15.2% glutaral, 19.7% (ethylendioxy) dimethanol,	3%	60	5.7 ± 0.5
Glutaraldehyde*		25% glutaraldehyde	2%	60	4.0 ± 0.7
Sekusept™ aktiv	Oxidizer	> 1.000 ppm peracetic acid (PAA)	2%	240	2.0 ± 0.3
NaOH*	Base	Sodium hydroxide	0.05 M	15	5.9 ± 0.2
			1 M	15	6.5 ± 0.7

## Discussion

After the phase I/II clinical trials, the next step is to prepare the drug product for phase III trials and market release. For this, we performed real-time experiments while developing the process for storage stability studies. Hold-steps were identified for the downstream process with product stability up to 3 months at 4 °C and up to 5 years at −20 °C in the drug product. The hold-steps make it possible to establish a more flexible downstream and packaging schedule. In addition, they are suitable as in-process controls to show production consistency but also to test bulk drug substance, as recommended by regulatory authorities. However, stability needs to be shown in large-scale production under GMP conditions too, and cumulative product age must be accounted for when hold-steps are indeed used during manufacturing. Sample analysis also needs to be extended, i.e., sterility, bioburden, identity, purity, and quality, to include not only virus titer, but also a parameter profile indicating stability (World-Health-Organization 2011; Food-and-Drug-Administration 2020). Overall, the results of our experiments support the proof-of-concept and robustness of this novel production process. Stability data at ≤ −65 °C for up to 7 years of clinical batches under GMP conditions emphasizes the suitability of the DP-F formulation for long-term storage.

Furthermore, we demonstrated H-1PV stability by employing stress tests with UV irradiation as well as temperature and pH range. Our findings show that H-1PV in DP-F is extremely stable against UV irradiation. H-1 virus in DP-i.v. containing only 10% Iodixanol in Visipaque/Ringer is more susceptible to UV irradiation, demonstrating a reduction in infectious titer of approximately 1 log_10_. However, this susceptibility can be avoided by using light-protective packaging. Moreover, H-1PV in H-1PV-RIN and H-1PV-VTE is reduced by over 4 log_10_ due to UV irradiation as was previously shown by Angelova et al. (2009). These results confirm that Iodixanol protects H-1PV from UV irradiation. This knowledge could be also applied to other virus-based drugs. The stability at pH ≥ 1.25 suggests that H-1PV might possibly even be stable in an acidic stomach environment after oral administration. Additionally, H-1PV in DP-F formulation is stable up to 72 h at 37 °C and up to 24 h at 50 °C whereas DP-i.v. is stable up to 24 h at both 37 °C and 50 °C. This significantly reduces the risk of drug titer loss by unintentional variations in transport conditions. We also examined the effects of lyophilization simulation by dehydration and reconstitution of H-1PV in DP-F. This could offer a great advantage for transport and storage, independent of cooling systems at ambient temperature. Our simulation demonstrated that different doses up to 5E9 PFU/ml of H-1PV were stable after reconstitution. Similar findings were reported for porcine parvovirus whereas infectious titers of Maus Elberfeld virus and vesicular stomatitis virus were reduced after lyophilization (Uhlenhaut et al. 2005). To successfully apply lyophilization, further testing with industrial grade lyophilization devices are needed, followed by process validation (Jameel et al. 2021a; Jameel et al. 2021b).

In-use stability for 4 days at RT and the inertness to the surface of the injection sets make the processes for ParvOryx preparation and administration more flexible. This guarantees that patients receive the required dose during clinical trials. The 4-day hold-up time between dose preparation at the pharmacy and drug administration was chosen so that the drug injections could be prepared on working days for administration over the weekend. This time interval also makes it possible to prepare up-front up to 4 daily injections at the same time.

In addition to the stability of the H-1PV drug product, deactivation for safety reasons at all facilities, including manufacturer, pharmacy, clinic, and transport vehicle, must be guaranteed. This is important to avoid contamination of the environment and all contact persons. Parvoviruses are believed to demonstrate a very low susceptibility to physico-chemical treatments (European-Medicines-Agency 1997). According to the RKI, a 4 log_10_ reduction in infectious titer, at which virus is inactivated to 99.99%, is considered to provide sufficient virucidal activity of the disinfection solution (Schwebke and Rabenau 2012). In case of GMP produced H-1PV with infectious titer of maximum 5E9 PFU/ml, the 4 log_10_ reduction in infectious titer would result in virus titer of 5E5 PFU/ml. Geletneky et al. (2017) demonstrated that with a total dose of 1E6 PFU applied intratumorally no immune response or virus shedding was observed. Physical virus inactivation usually employs heat inactivation or UV treatment (Patterson et al. 2020). In our assessment of four different physical deactivation methods, we were able to demonstrate that heat deactivation at 99 °C for 10 min and autoclavation at 121 °C for 20 min achieves sufficient H-1PV inactivation with an over 5 log_10_ reduction in the infectious titer. However, H-1PV inactivation by using UV irradiation is formulation dependent as Iodixanol protects H-1PV. In order to inactivate H-1PV in DP-F (48% Iodixanol) and in DP-i.v. (10% Iodixanol) with UV irradiation, the H-1PV solution must be diluted beforehand with aqueous solutions. H-1PV infectious titer can also be reduced by nanofiltration; however, only approximately 3.4 log_10_ could be depleted using this method. Therefore, autoclavation is the method of choice for physical inactivation and can be used for instruments, waste and to pretreat glass, and solutions. For small volumes heat deactivation is a good alternative for a rapid virus deactivation at research facilities. All chemical disinfectants that we used in our H-1PV inactivation experiments are currently recommended by the RKI (Robert-Koch-Institut 2017, 2022) and comply with the hygiene plan at DKFZ. In our analysis of chemical disinfectants, we demonstrated that ethanol-based hand disinfectants are ineffective for inactivating H-1PV, deactivating only 0.5 log_10_. Low susceptibility of parvoviruses to ethanol has been previously reported (Eterpi et al. 2009; Zhou 2022). Therefore, wearing gloves is a necessary precaution measure to protect the hands while working with H-1PV. In contrast, aldehyde-based solutions containing glutaral, formaldehyde, or glutaraldehyde used for instruments and surface disinfection can inactivate H-1PV quite effectively. Our assessment of four aldehyde-based disinfectants that are recommended by the RKI showed that Lysoformin^®^ containing formaldehyde and glutaral is least effective for inactivating H-1PV, with a ~3 log_10_ reduction in infectious titer. Incidin™ Rapid, which is aldehyde- and oxidizer-based, demonstrated formulation-dependent inactivation properties, being unable to sufficiently deactivate H-1PV in DP-F and DP-i.v. H-1PV formulations, but inactivating H-1PV in Ringer solution up to 4.8 log_10_ and in VTE formulation up to 5.9 log_10_. If Incidin™ Rapid is used with DP-F and in DP-i.v. formulations, the formulations should be first diluted with aqueous solutions. Furthermore, 2% glutaraldehyde solution is quite effective for inactivating infectious titer by 4 log_10_ in DP-F formulation while preserving the morphology of the virus for ELMI analysis (Rodgers et al. 1985). Moreover, 3% Korsolex^®^ basic, a glutaral-containing disinfectant, demonstrated superior inactivation results, reducing infectious titer 5.7 log_10_ in the DP-F formulation. Peracetic acid (PAA)-based Sekusept™ aktiv with high oxidative characteristics demonstrated results similar to those for Incidin™ Rapid by being able to deactivate H-1PV in H-1PV-RIN and H-1PV-VTE formulations by >6 log_10_ but demonstrating only 2 log_10_ infectious titer reduction in DP-F and DP-i.v. formulations. Inactivation of porcine parvovirus (PPV) and minute virus of mice (MVM) parvovirus with PAA-based compounds was previously reported by Eterpi et al. (2009). Sekusept™ aktiv has the advantage of being pH-neutral and, therefore, safer for medical staff and patients as well as being more suitable for different instruments and surfaces than other tested disinfectants. A noncommercial solution of sodium hydroxide demonstrated sufficient disinfection results, being able to reduce infectious virus titer 5.9 (0.05 M) or 6.5 (1 M) log_10_ after incubation for 15 min. The times recommended by manufacturers for CIP procedures are at least 30 min; therefore, we can expect at least a 6 log_10_ reduction in infectious titer after 30 min. While being a part of the CIP procedure for many devices, such as for tangential flow filtration or chromatography column used during the H-1PV downstream process, it is also inexpensive. Therefore, NaOH can be utilized to clean these devices and to inactivate virus in the same step. Boschetti et al. (2003) demonstrated deactivation of MVM virus with NaOH concentrations of ≥0.1M after incubation for 1 min. However, NaOH is not recommended for disinfecting surfaces due to safety aspects and corrosive properties, even for a rapid and inexpensive deactivation. Taken together, we would recommend using Korsolex^®^ basic for surface and instrument disinfection in general (during manufacturing, transport, or storage or during preparation at the pharmacy or administration at the hospital) since it inactivates H-1PV in any formulation. Sekusept™ aktiv or Incidin™ Rapid could be used to deactivate aqueous solutions of H-1PV whereas solutions of glutaraldehyde or NaOH could be used to inactivate ELMI samples or for CIP procedures during the manufacturing process, respectively.

In summary, the present study shows the major role of a defined formulation. ParvOryx provides the stability and inactivation properties required for production and administration of such a drug. Indeed, stability and environmental safety key factors should already be considered in the beginning of the process development, including a proper hygiene plan. In our case 48% Iodixanol in Visipaque/Ringer stabilizes H-1PV during storage at different temperatures and reduces backflow during drug injection (Leuchs et al. 2016). Furthermore, 48% Iodixanol in Visipaque/Ringer has the same viscosity of around 4 mPa*s as blood at 37 °C (Rosenson et al. 1996) and is a contrast reagent, making it possible to observe target distribution in real time by computed tomography and resulting in precise local delivery (Leuchs et al. 2016). In this formulation no aggregations (Leuchs et al. 2017) and no adsorption to the injection sets that were used is observed. The DP-F formulation also protects the virus against UV irradiation and some disinfectants. For the drug product formulated in 48% Iodixanol in Visipaque/Ringer an optimal hygiene protocol including validated disinfection solutions should be used to inactivate H-1PV, as this formulation is extremely stable. However, the robust stability of this formulation facilitates the storage, transport, and use of the drug for oncolytic virotherapy.

## Supplementary information

ESM 1: Table S1 and Figure S1 (PDF 90 kb)

## Data Availability

The datasets generated and analyzed during the current study are available from the corresponding author on reasonable request.
