# Supplementary material for: Stability and safety key factors of the oncolytic protoparvovirus H-1 from manufacturing to human application
Source: Appl Microbiol Biotechnol. 2023 May 20;107(15):4777–87. doi: 10.1007/s00253-023-12521-4 (PMC10345020; doi:10.1007/s00253-023-12521-4)
Supplement: Supplementary file 1 — Table S1 and Figure S1 (PDF 90 kb) [file 253_2023_12521_MOESM1_ESM.pdf]

Journal:

Applied Microbiology and Biotechnology

Title:

Key factors regarding stability and environmental safety of the oncolytic protoparvovirus H-1 from manufacturing to human application

Veronika Frehtman, Daniel Wohlfarth, Marcus Müller, Ottheinz Krebs, Barbara Leuchs

Affiliation:

German Cancer Research Center, Tumor Virology, Im Neuenheimer Feld 280, 69120 Heidelberg, Germany

Corresponding author:

Barbara Leuchs

German Cancer Research Center

Tumor Virology F010

Im Neuenheimer Feld 280

69120 Heidelberg

Germany

B.Leuchs@dkfz.de; 00496221424300; Fax: 00496221424301

Table S1: H-1PV antibody response in blood serum of people working with the H-1 virus for many years via hemagglutination inhibition assay performed at Labor Enders, Germany

Staff:	2010/2011	2019
TA: 20 years	LLOQ	LLOQ
TA: 20 years	LLOQ	LLOQ
TA: 11 years	LLOQ	LLOQ
TA: 4 years	n.a.	LLOQ
MSc: 5 years	n.a.	LLOQ
Leader: 20 years	LLOQ	LLOQ
study nurse	n.a.	LLOQ

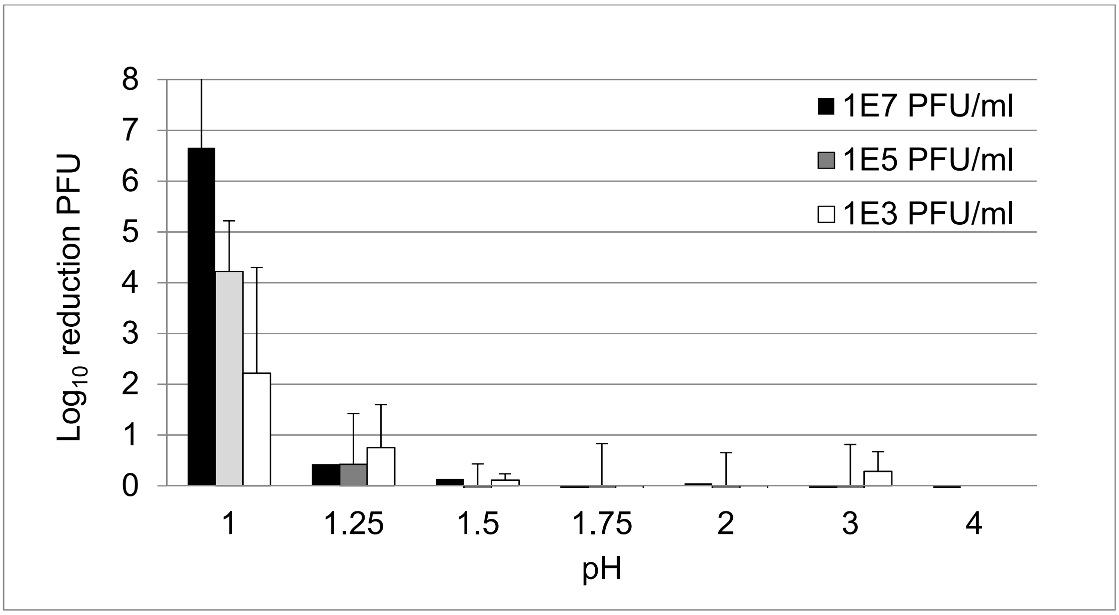

Fig. S1 pH stability of H-1PV in DP-F with 3 different titers [1E7 PFU/ml, 1E5 PFU/ml and 1E3 PFU/ml] at pH of 1, 1.25, 1.5, 1.75, 2, 3, and 4 adjusted with HCl for 30 min. After 30 min the H-1PV/HCl solution was neutralized with VP-SFM™ medium with 5% FBS and immediately analyzed
